# Associations of fear of recurrence and social support with post-traumatic stress disorder and disease recurrence in patients with severe acute pancreatitis: a retrospective study

**DOI:** 10.3389/fmed.2026.1893298

**Published:** 2026-08-19

**Authors:** Guofu Bai, Xiaoyan Gong, Chen Huang, Yiyu Zhuang

**Affiliations:** Nursing Department, Sir Run Run Shaw Hospital, Zhejiang University School of Medicine, Hangzhou, Zhejiang, China

**Keywords:** disease recurrence, fear of recurrence, post-traumatic stress disorder, severe acute pancreatitis, social support, subgroup analysis

## Abstract

**Objective:**

To explore the correlations among fear of recurrence, post-traumatic stress disorder (PTSD), social support and disease recurrence in patients discharged after severe acute pancreatitis (SAP), so as to provide evidence for psychological intervention and recurrence prevention in SAP survivors.

**Methods:**

A total of 855 adult inpatients newly diagnosed with SAP at Sir Run Run Shaw Hospital, Zhejiang University School of Medicine from January 2020 to April 2025 were retrospectively enrolled. Baseline clinical data and 12-month post-discharge follow-up data including SAP recurrence events and psychological scale scores were collected. Intergroup comparison, correlation analysis, multivariate Logistic regression, linear regression and mediating effect model analyses were performed for statistical analysis.

**Results:**

The patients were divided into the recurrence group (144 cases) and non-recurrence group (711 cases). Baseline demographic characteristics, past medical history, etiology and disease severity were comparable between the two groups, except that the recurrence group had a significantly higher ICU admission rate (*p* = 0.026). The recurrence group presented significantly higher Fear of Progression Questionnaire-Short Form (FoP-Q-SF) and PTSD self-rating scale (PTSD-SS) scores, higher PTSD prevalence and severity, and lower Perceived Social Support Scale (PSSS) scores and proportion of high social support (all *p* < 0.001). Fear of recurrence was positively correlated with PTSD-SS scores (*ρ* = 0.64, *p* < 0.001). Multivariate regression analysis confirmed that fear of disease recurrence was positively associated with post-traumatic stress disorder (PTSD), while higher levels of social support were negatively associated with PTSD. PTSD-SS scores were positively associated with acute pancreatitis recurrence. Mediating effect analysis showed that PTSD had no significant mediating effect between social support and recurrence, nor between fear of recurrence and recurrence (all *p* > 0.05).

**Conclusion:**

Fear of recurrence is positively associated with PTSD symptoms in SAP patients, while high social support is negatively associated with this adverse psychological impact via a buffering interaction effect. PTSD severity is independently associated with an increased risk of SAP recurrence. These findings highlight the importance of psychological assessment and social support interventions in the long-term management of SAP survivors.

## 1 Introduction

Severe Acute Pancreatitis (SAP) is a common critical care emergency of the digestive system, characterized clinically by rapid onset, rapid progression, multiple complications, and a high mortality rate (1–4). In China, the incidence of acute pancreatitis has increased in recent decades, with reported annual incidence rates ranging from 13 to 45 cases per 100,000 population (5). Approximately 20% of patients with acute pancreatitis develop moderately severe or severe disease, with reported mortality rates ranging from 13 to 35% (6). In recent years, with the continuous improvement of intensive care and comprehensive treatment techniques, the in-hospital survival rate of SAP patients has improved significantly. However, a substantial body of research indicates that after discharge, SAP patients face multiple adverse long-term outcomes, including pancreatic exocrine and endocrine insufficiency, high recurrence and readmission rates, and progression to chronic pancreatitis (7, 8). These outcomes not only impose a heavy medical and economic burden but also severely impair patients’ quality of life. Currently, clinical research on SAP recurrence has predominantly focused on physiological and lifestyle risk factors such as biliary tract disease, hyperlipidemia, alcohol consumption, and improper diet, while the exploration of psychosocial factors remains relatively scarce (9, 10).

Critical illnesses are highly likely to cause persistent psychological trauma in patients, and Post-traumatic Stress Disorder (PTSD) is the most common psychological sequela among survivors of critical illness (11). During the course of diagnosis and treatment, SAP patients endure intensely painful experiences, including severe abdominal pain, invasive procedures, and isolation in the intensive care unit. These powerful traumatic stimuli can readily lead to PTSD symptoms after discharge, such as recurrent traumatic memories, anxiety, insomnia, and emotional depression (12–14). In addition to PTSD, anxiety and depressive symptoms are also common psychological complications among patients recovering from acute pancreatitis, with reported prevalence rates of 33.4 and 36.8%, respectively (15). These psychological symptoms have been shown to negatively affect health-related quality of life during the recovery period (16). Existing research has confirmed that PTSD can disrupt the body’s neuro-endocrine and immune balance (17, 18), induce a state of chronic inflammation that is detrimental to organ function recovery, and simultaneously promote unhealthy lifestyle behaviors (19), thereby indirectly increasing the risk of chronic disease recurrence. At this stage, research on SAP has largely focused on in-hospital treatment and short-term physiological indicators, with insufficient attention given to the long-term negative impacts of psychological trauma, such as PTSD, following discharge.

Fear of recurrence refers to the persistent fear and anxiety patients experience regarding the potential return of their disease (20), and it is prevalent among individuals recovering from various digestive, oncological, and chronic critical illnesses (21, 22). Long-term, excessive fear of recurrence can exacerbate a patient’s psychological stress response, creating a vicious cycle that intensifies psychological distress (23), while also inducing high-risk behaviors such as unhealthy eating and disrupted sleep patterns (24), further increasing the likelihood of disease recurrence. In contrast, social support can mitigate disease-related psychological distress, enhance adaptive coping strategies, and reduce the adoption of unhealthy behaviors (25), thereby potentially attenuating the adverse impact of negative emotions on clinical outcomes (26). Studies have demonstrated that fear of recurrence can play a mediating role between PTSD and adverse disease outcomes, serving as a crucial link connecting psychological stress and disease recurrence (27). However, no study has yet explicitly investigated the inter-relationships among fear of recurrence, PTSD, social support, and disease recurrence in patients with SAP.

Therefore, this study aims to explore the correlations among fear of recurrence, PTSD, social support and disease recurrence in patients discharged after SAP. This research aims to provide a reference for implementing psychological screening for discharged patients, reducing disease recurrence rates, and improving the long-term physical and mental health outcomes of this population.

## 2 Methods

### 2.1 Data source and study population

**Figure 1:**
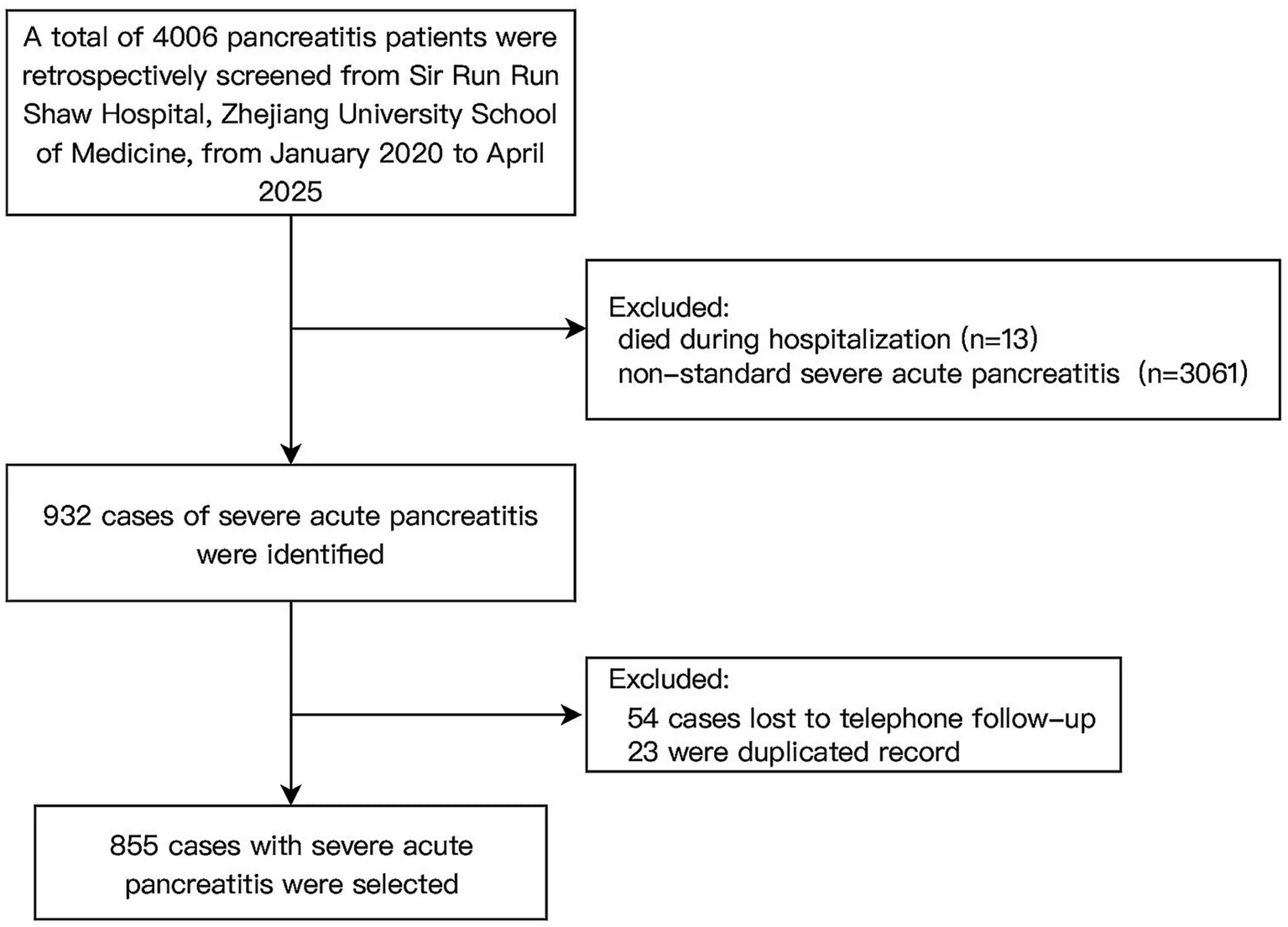
Flowchart of patient enrollment and study population selection. A total of 4,006 patients with pancreatitis were initially screened. After applying the exclusion criteria, 855 patients with severe acute pancreatitis were included in the final analysis.

Clinical data of adult inpatients first diagnosed with severe acute pancreatitis between January 2020 and April 2025 at Sir Run Run Shaw Hospital, Zhejiang University School of Medicine, were retrospectively collected, ans structured post-discharge follow-up assessments were conducted to obtain psychological questionnaire scores and record acute pancreatitis recurrence events. SAP was diagnosed according to the 2012 Revised Atlanta Classification (28). The exclusion criteria for this study were as follows: patients who died during hospitalization due to severe acute pancreatitis or its complications; patients with a prior confirmed diagnosis of chronic pancreatitis, pancreatic cancer, or other organic pancreatic diseases; patients with a prior history of mental illness (e.g., schizophrenia, bipolar disorder) or a confirmed diagnosis of PTSD; patients with severe cognitive impairment (e.g., Alzheimer’s disease, cognitive impairment following traumatic brain injury) rendering them unable to complete the PTSD assessment questionnaires; patients with severely missing follow-up data or a follow-up period of less than 12 months, making it impossible to meet the analytical requirements for the study variables; and patients lost to follow-up. The detailed flowchart is shown in Figure 1. This study was approved by the Ethics Committee of Sir Run Run Shaw Hospital, Zhejiang University School of Medicine (Ethics Approval No.: Run Run Shaw Hospital Ethics Review 2026 Research No. 0351). Written informed consent was obtained from all enrolled patients. This retrospective observational study was reported in accordance with the STROBE guidelines.

### 2.2 Data collection

Patient admission data were extracted from the hospital’s electronic medical record system. Demographic characteristics: sex, marital status (missing, widowed, married, unmarried, divorced), age, use of psychotropic drugs, alcohol cessation status (never drinker, former drinker, current drinker, heavy drinker), smoking cessation status (never smoker, former smoker, current smoker), career (employed, unemployed, retired), and place of residence (rural, urban); past medical history: hypertension, diabetes mellitus, hyperlipidemia, coronary heart disease, cancer, history of surgery, and history of major diseases (e.g., acute myocardial infarction, cardiogenic shock, stroke, major trauma); etiology of SAP (biliary, alcoholic, hypertriglyceridemic, other); disease severity indicators: organ failure (ARDS, acute kidney injury, acute liver failure, shock, heart failure, etc.), intensive care unit (ICU) admission, invasive mechanical ventilation, and surgical treatment; follow-up data: Information was collected for the 12-month period following discharge through the hospital outpatient system, telephone follow-up, and interviews with patients’ family members. Psychological assessments were completed by patients themselves, whereas family interviews were conducted when necessary to supplement follow-up information, including the occurrence of acute pancreatitis recurrence, rehospitalization events, and other relevant clinical outcomes. This information included the occurrence of acute pancreatitis recurrence and the scores from follow-up assessment scales [Perceived Social Support Scale (PSSS), Fear of Progression Questionnaire-Short Form (FoP-Q-SF), and PTSD self-rating scale (PTSD-SS)]. These psychological assessment instruments have been widely used in clinical populations and have demonstrated satisfactory reliability and validity in Chinese populations, supporting their applicability for evaluating perceived social support, disease-related fear, and PTSD symptoms in the present study (29–31). The PSSS is a 12-item instrument to assess support from family, friends, relatives, and colleagues. The total score ranges from 12–84, with higher scores indicating higher perceived social support. A score between 12 and 36 indicates low support, 37–60 indicates medium support, and 61–84 indicates high support. The FoP-Q-SF score varies between 12 and 60 points, and the higher the score, the greater the fear of disease progression. The PTSD-SS scores of ≥50 indicate the presence of positive symptoms, while a score of ≥60 suggests severe PTSD. Higher scores correspond to greater severity of PTSD. A 5-point Likert scale was used to assess patients’ fear of acute pancreatitis recurrence (0 = no fear, 4 = extreme fear).

### 2.3 Outcome

The outcome measure was defined as acute pancreatitis recurrence, re-hospitalization due to acute pancreatitis within 12 months after discharge, and the time to recurrence was recorded.

### 2.4 Sample size calculation

Sample size was calculated based on the primary outcome of acute pancreatitis recurrence within 12 months after discharge and PTSD status as the primary exposure factor. Assuming a two-sided *α* level of 0.05, a power of 80%, a PTSD prevalence of 30%, a recurrence rate of 17% in the non-PTSD group, and an expected odds ratio (OR) of 1.80, the required sample size was estimated to be 682 patients with complete outcome data. Considering approximately 15% potential loss to follow-up and incomplete data, the final required sample size was estimated at approximately 803 patients.

### 2.5 Statistical analysis

Statistical analysis was performed using R software (version 4.3.3). Normally distributed continuous data were presented as mean ± standard deviation (SD), while skewed continuous data were presented as median (interquartile range). Categorical data were presented as frequency (percentage). For intergroup comparisons, independent samples t-tests were used for normally distributed data, Wilcoxon rank-sum tests for skewed data, and χ^2^ tests or Fisher’s exact tests for categorical data. Spearman’s rank correlation analysis was used to investigate the association between the degree of fear of recurrence and PTSD-SS score. Significance testing was determined using the asymptotic normal approximation.

We established univariate and multivariate logistic regression or linear regression models to explore the relationships among social support, fear of recurrence and PTSD, and calculated OR, beta coefficients (*β*) and 95% confidence intervals (95% CI). Variables with *p* < 0.05 in univariate analyses were entered into multivariate models. Multicollinearity among candidate variables was assessed using the variance inflation factor (VIF) and generalized variance inflation factor (GVIF). For variables with multiple degrees of freedom, the adjusted GVIF ( $\mathrm{GVI}F^{1/(2\mathrm{df})})$ was calculated. A VIF value <5 or an adjusted GVIF value <2.24 was considered acceptable, indicating the absence of substantial multicollinearity. Interaction models were further constructed in the multivariate analyses to examine the interactive effect between fear of recurrence and PSSS scores. Logistic regression was used to analyze the associations of PTSD, social support and fear of recurrence with disease recurrence, as well as the moderating effect of PTSD. Mediation models were built to assess the mediating role of PTSD in the relationships between fear of recurrence and disease recurrence, and between social support and disease recurrence. All statistical tests in this study were two-sided, and *p* < 0.05 was considered statistically significant.

## 3 Results

### 3.1 Patient characteristics

A total of 4,006 patients with pancreatitis were initially screened between January 2020 and April 2025. After applying the predefined eligibility criteria and excluding ineligible cases, in-hospital deaths, duplicate records, and patients lost to follow-up, 855 patients with SAP were finally included in the analysis (Figure 1). Baseline characteristics are presented in Table 1. All participants were divided into the recurrence group (144 cases, 16.84%) and the non-recurrence group (711 cases, 83.16%) according to the recurrence of SAP. There were no statistically significant differences between the two groups in demographic characteristics, past medical history, etiology, or disease severity, except that the ICU admission was significantly higher in the recurrence group than in the non-recurrence group (*p* = 0.026). All other differences were not statistically significant (all *p* > 0.05). The recurrence group had significantly higher FoP-Q-SF scores, PTSD-SS scores, and a higher prevalence and severity of PTSD, but significantly lower PSSS scores and a lower proportion of high social support than the non-recurrence group (all *p* < 0.001).

**Table 1:** Comparison of baseline clinical characteristics of severe acute pancreatitis patients with and without recurrence.

Characteristic	Overall, *N* = 855	Non-recurrence *N* = 711	Recurrence, *N* = 144	*p*-value^a^
Sex				0.925
Female	276 (32.28%)	230 (32.35%)	46 (31.94%)	
Male	579 (67.72%)	481 (67.65%)	98 (68.06%)	
Marital status				0.639
Missing	9 (1.05%)	6 (0.84%)	3 (2.08%)	
Widowed	18 (2.11%)	16 (2.25%)	2 (1.39%)	
Married	685 (80.12%)	571 (80.31%)	114 (79.17%)	
Unmarried	109 (12.75%)	89 (12.52%)	20 (13.89%)	
Divorced	34 (3.98%)	29 (4.08%)	5 (3.47%)	
**Age**	46.00 (35.00, 60.00)	46.00 (35.00, 59.00)	48.50 (35.00, 64.00)	0.770
Education				0.882
Primary school or below	227 (26.55%)	186 (26.16%)	41 (28.47%)	
Middle or high school	366 (42.81%)	304 (42.76%)	62 (43.06%)	
College or undergraduate	247 (28.89%)	208 (29.25%)	39 (27.08%)	
Master’s degree or above	10 (1.17%)	8 (1.13%)	2 (1.39%)	
Missing	5 (0.58%)	5 (0.70%)	0 (0.00%)	
Use of psychotropic drugs				0.597
No	849 (99.30%)	705 (99.16%)	144 (100.00%)	
Yes	6 (0.70%)	6 (0.84%)	0 (0.00%)	
Alcohol cessation status				0.615
Never drinker	486 (56.84%)	398 (55.98%)	88 (61.11%)	
Former drinker	81 (9.47%)	71 (9.99%)	10 (6.94%)	
Current drinker	280 (32.75%)	235 (33.05%)	45 (31.25%)	
Heavy drinker	8 (0.94%)	7 (0.98%)	1 (0.69%)	
Smoking cessation status				0.103
Never smoker	501 (58.60%)	408 (57.38%)	93 (64.58%)	
Former smoker	77 (9.01%)	70 (9.85%)	7 (4.86%)	
Current smoker	277 (32.40%)	233 (32.77%)	44 (30.56%)	
Career				0.673
Employed	220 (25.73%)	183 (25.74%)	37 (25.69%)	
Unemployed	540 (63.16%)	452 (63.57%)	88 (61.11%)	
Retired	95 (11.11%)	76 (10.69%)	19 (13.19%)	
Place of residence				0.405
Urban	448 (52.40%)	368 (51.76%)	80 (55.56%)	
Rural	407 (47.60%)	343 (48.24%)	64 (44.44%)	
Hypertension				0.881
No	637 (74.50%)	529 (74.40%)	108 (75.00%)	
Yes	218 (25.50%)	182 (25.60%)	36 (25.00%)	
Diabetes mellitus				0.747
No	590 (69.01%)	489 (68.78%)	101 (70.14%)	
Yes	265 (30.99%)	222 (31.22%)	43 (29.86%)	
Hyperlipidemia				0.194
No	634 (74.15%)	521 (73.28%)	113 (78.47%)	
Yes	221 (25.85%)	190 (26.72%)	31 (21.53%)	
Coronary heart disease				0.755
No	836 (97.78%)	694 (97.61%)	142 (98.61%)	
Yes	19 (2.22%)	17 (2.39%)	2 (1.39%)	
Cancer				0.055
No	837 (97.89%)	693 (97.47%)	144 (100.00%)	
Yes	18 (2.11%)	18 (2.53%)	0 (0.00%)	
History of surgery				0.430
No	649 (75.91%)	536 (75.39%)	113 (78.47%)	
Yes	206 (24.09%)	175 (24.61%)	31 (21.53%)	
History of major diseases				0.202
No	821 (96.02%)	680 (95.64%)	141 (97.92%)	
Yes	34 (3.98%)	31 (4.36%)	3 (2.08%)	
Etiology				0.801
Alcoholic	18 (2.11%)	16 (2.25%)	2 (1.39%)	
Biliary	185 (21.64%)	149 (20.96%)	36 (25.00%)	
Hypertriglyceridemic	346 (40.47%)	288 (40.51%)	58 (40.28%)	
Missing	4 (0.47%)	4 (0.56%)	0 (0.00%)	
Other	302 (35.32%)	254 (35.72%)	48 (33.33%)	
Organ failure				0.812
No	530 (61.99%)	442 (62.17%)	88 (61.11%)	
Yes	325 (38.01%)	269 (37.83%)	56 (38.89%)	
ICU admission				0.026
No	164 (19.18%)	146 (20.53%)	18 (12.50%)	
Yes	691 (80.82%)	565 (79.47%)	126 (87.50%)	
Invasive mechanical ventilation				0.714
No	628 (73.45%)	524 (73.70%)	104 (72.22%)	
Yes	227 (26.55%)	187 (26.30%)	40 (27.78%)	
Surgical treatment				0.276
No	196 (22.92%)	168 (23.63%)	28 (19.44%)	
Yes	659 (77.08%)	543 (76.37%)	116 (80.56%)	
**FoP-Q-SF score**	28.00 (23.00, 46.00)	27.00 (23.00, 40.00)	45.00 (24.00, 49.00)	<0.001
Fear of recurrence				<0.001
0	308 (36.02%)	278 (39.10%)	30 (20.83%)	
1	296 (34.62%)	260 (36.57%)	36 (25.00%)	
3	127 (14.85%)	88 (12.38%)	39 (27.08%)	
4	124 (14.50%)	85 (11.95%)	39 (27.08%)	
**PSSS score**	58.00 (37.00, 69.00)	61.00 (41.00, 70.00)	38.00 (31.00, 65.00)	<0.001
Perceived social support				<0.001
High support	599 (70.06%)	533 (74.96%)	66 (45.83%)	
Low mid support	256 (29.94%)	178 (25.04%)	78 (54.17%)	
**PTSD-SS score**	65.00 (44.00, 98.00)	61.00 (43.00, 90.00)	97.00 (60.50, 102.00)	<0.001
PTSD				<0.001
No PTSD	596 (69.71%)	531 (74.68%)	65 (45.14%)	
PTSD	259 (30.29%)	180 (25.32%)	79 (54.86%)	
PTSD severity				<0.001
Mild PTSD	8 (0.94%)	7 (0.98%)	1 (0.69%)	
Moderate to severe PTSD	251 (29.36%)	173 (24.33%)	78 (54.17%)	
No PTSD	596 (69.71%)	531 (74.68%)	65 (45.14%)	

a Fisher’s exact test; Wilcoxon rank sum test; Pearson’s Chi-squared test; History of serious diseases including Acute myocardial infarction, cardiogenic shock, stroke, major trauma; PTSD, Post-traumatic Stress Disorder; ICU, Intensive Care Unit; PSSS, Perceived Social Support Scale; FoP-Q-SF, Fear of Progression Questionnaire-Short Form; PTSD-SS, Post-traumatic Stress Disorder self-rating scale. Bold *p*-values indicate the overall *p*-values for comparisons between the non-recurrence and recurrence groups.

### 3.2 Correlation of fear of recurrence on PTSD in SAP patients

**Figure 2:**
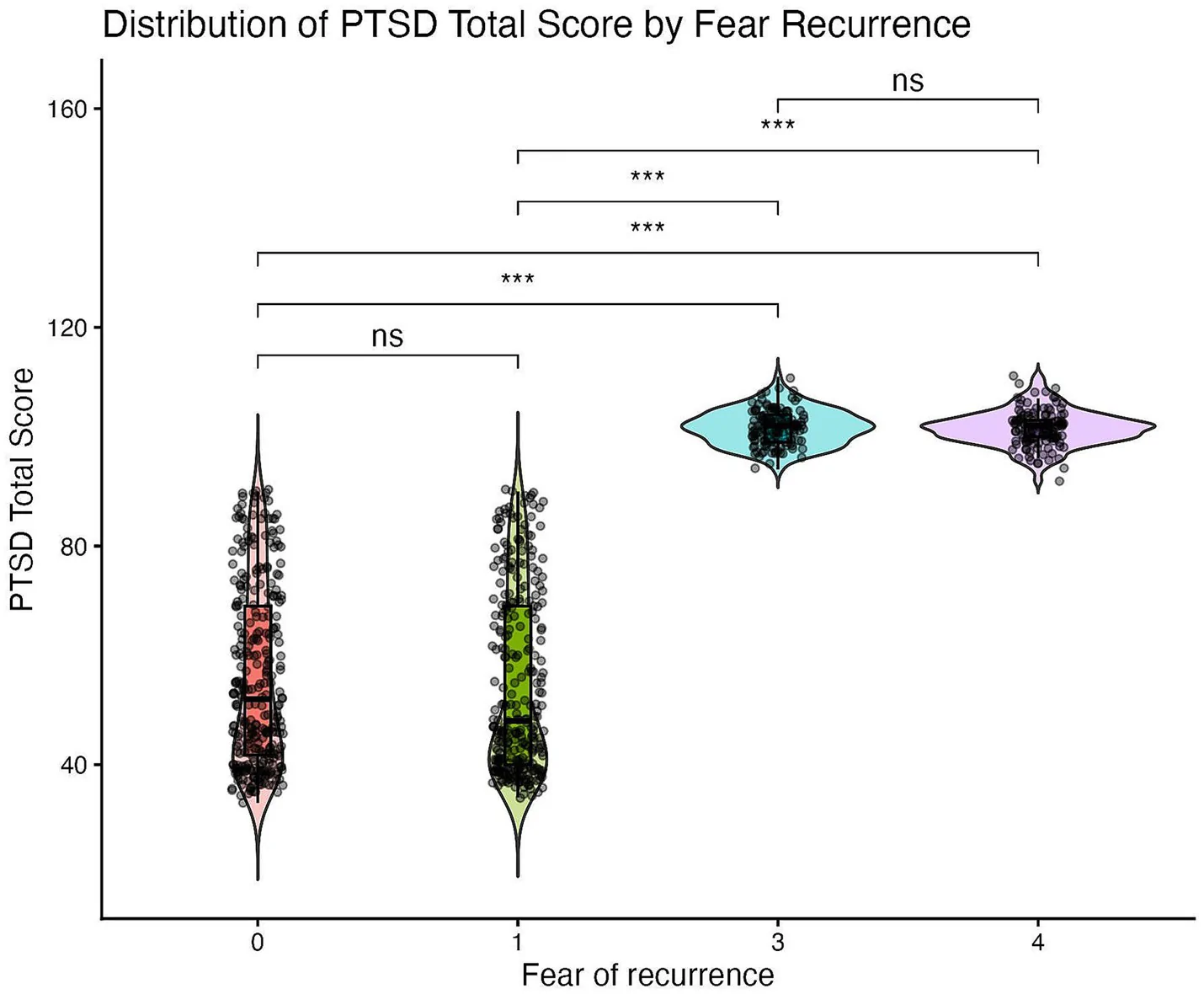
Distribution of PTSD-SS scores across different levels of fear of recurrence in patients with severe acute pancreatitis. PTSD-SS scores increased with increasing levels of fear of recurrence. Data are presented as median with interquartile range. ***p < 0.001; ns, not significant. PTSD-SS, post-traumatic stress disorder self-rating scale.

**Figure 3:**
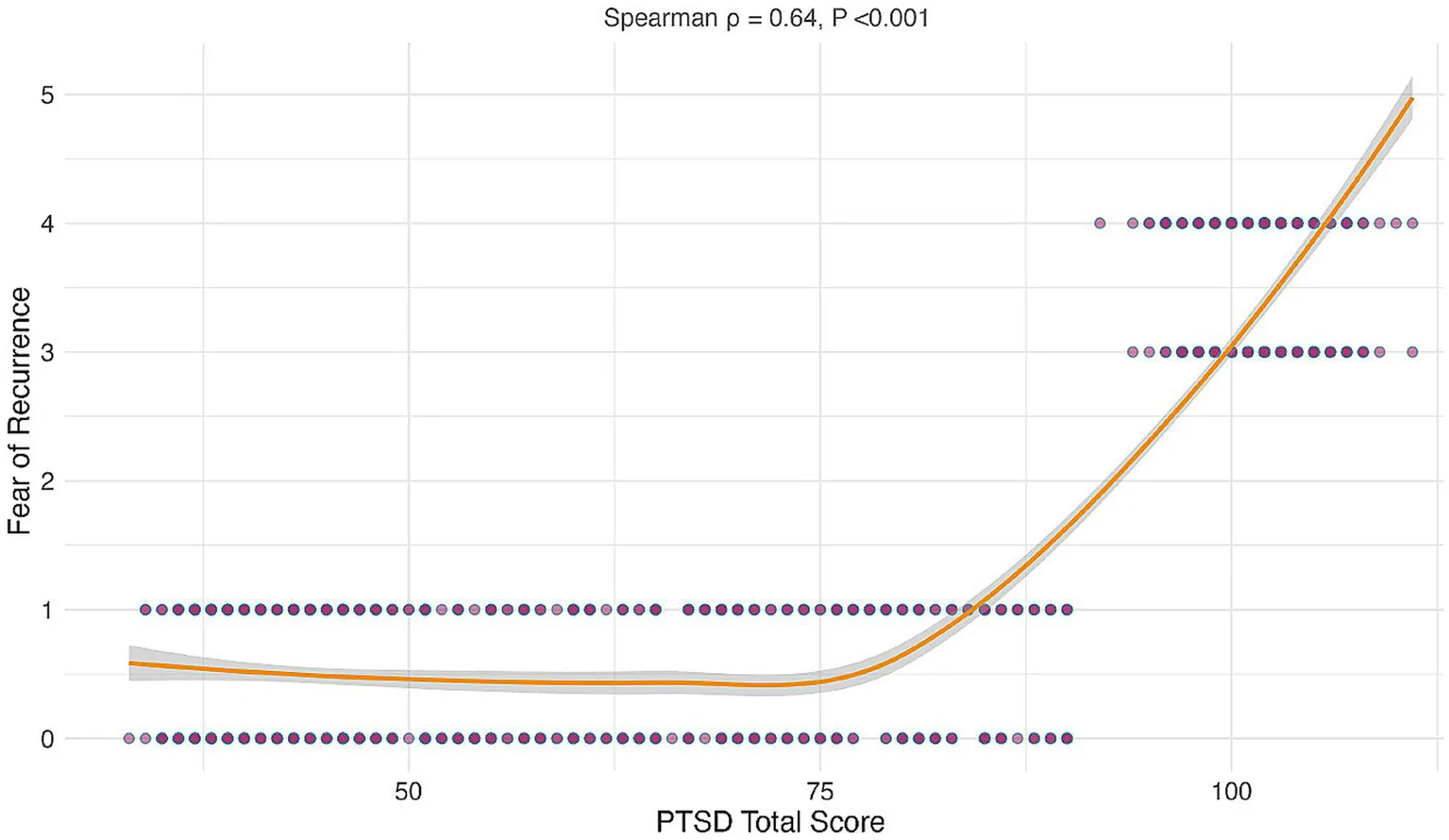
Correlation between PTSD-SS scores and fear of recurrence in patients with severe acute pancreatitis. The association between PTSD-SS scores and fear of recurrence was evaluated using Spearman’s rank correlation analysis. The solid line represents the fitted trend, and the shaded area indicates the 95% confidence interval. *ρ*, Spearman correlation coefficient; PTSD-SS, Post-traumatic Stress Disorder Self-rating Scale.

Non-parametric pairwise comparison revealed that patients with no fear of recurrence (0 point) and mild fear of recurrence (1 point) had relatively low PTSD-SS score with no significant intergroup difference (*p* > 0.05). Patients with moderate (3 point) and extreme fear of recurrence (4 point) presented significantly elevated PTSD-SS score, which were remarkably higher than those in the low-fear groups (0,1 point) (all *p* < 0.001) (Figure 2). Spearman correlation analysis demonstrated that the degree of fear of recurrence was positively correlated with the PTSD-SS score in discharged SAP patients (*ρ* = 0.64, *p* < 0.001) (Figure 3).

### 3.3 Associations and interactive effects of fear of recurrence and social support with PTSD in SAP patients

Univariate analysis showed that fear of recurrence, social support level, PSSS score, and FoP-Q-SF score were significantly correlated with PTSD occurrence and PTSD-SS score (all *p* < 0.001) (Supplementary Tables 1, 2). Collinearity diagnostics were performed before multivariate analysis, and variables with a VIF < 5 were considered eligible for inclusion in the multivariate models (Supplementary Tables 3, 4). Multivariate analysis demonstrated that the level of fear of recurrence was positively correlated with PTSD (OR = 3.67, 95%CI: 1.35–12.4, *p* = 0.019). Compared with patients with low-to-moderate social support, individuals with high social support had a 95% reduction in the risk of PTSD (OR = 0.05, 95%CI: 0.01–0.45, *p* = 0.002) (Supplementary Table 5). After adjusting for confounding variables, the interaction term between fear of recurrence and PSSS scores was negatively associated with PTSD (OR = 0.889, 95% CI 0.805–0.957; *p* = 0.005) (Supplementary Table 6). For the PTSD-SS score, high social support was negatively correlated with PTSD-SS scores (*β* = −0.500, 95% CI: −0.604 to −0.397; *p* < 0.001). FoP-Q-SF score was positively associated with higher PTSD-SS scores (*β* = 1.052, 95% CI: 0.904 to 1.200; *p* < 0.001) (Supplementary Table 7).

### 3.4 Effect of PTSD on recurrence and mediating effect analysis in SAP patients

**Figure 4:**
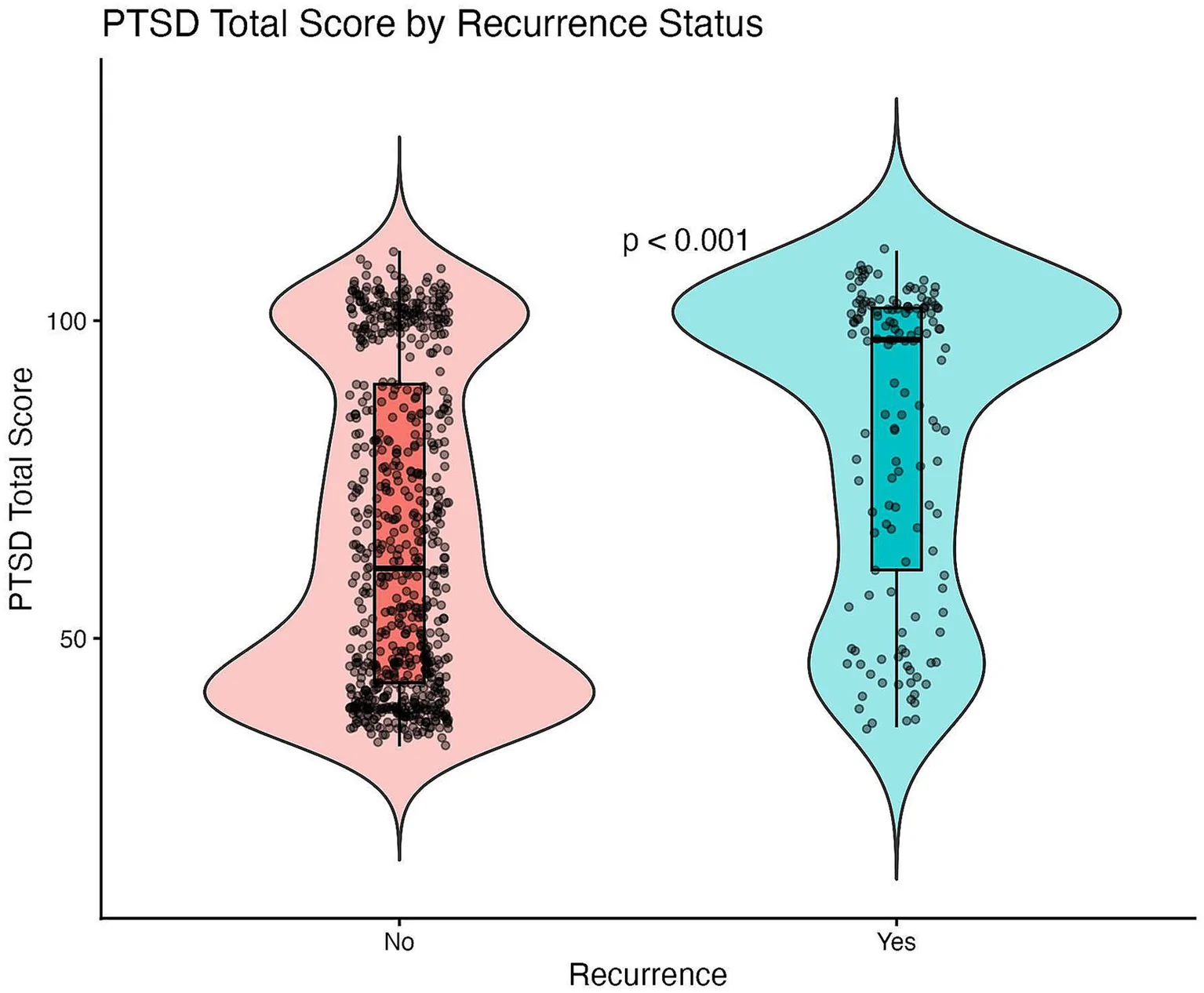
Distribution of PTSD-SS scores according to recurrence status in patients with severe acute pancreatitis. PTSD-SS scores were compared between patients with and without recurrence. Data are presented as violin plots with individual data points and boxplots. PTSD-SS, post-traumatic stress disorder self-rating scale.

**Figure 5:**
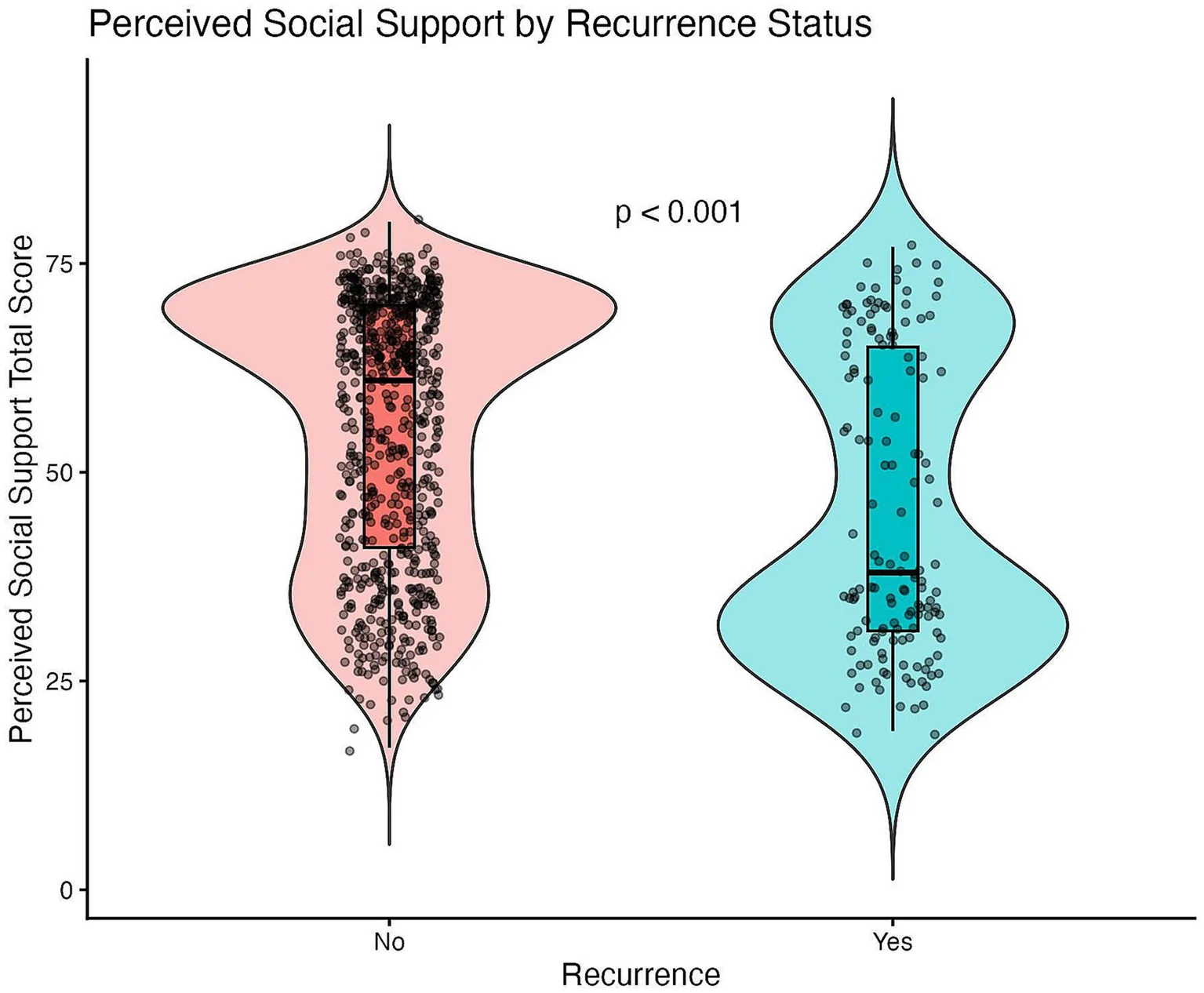
Distribution of perceived social support scores according to recurrence status in patients with severe acute pancreatitis. Perceived social support scores were compared between patients with and without recurrence. Data are presented as violin plots with individual data points and boxplots. PSSS, Perceived Social Support Scale.

Non-parametric pairwise comparison demonstrated that the recurrence group had a significantly higher PTSD-SS score and a lower PSSS score than the non-recurrence group (*p* < 0.001) (Figures 4, 5).

Univariate Logistic regression was used to analyze the correlation between various variables and recurrence. The results indicated that cancer, ICU admission, fear of recurrence, social support level, FoP-Q-SF score, PTSD-SS score, and PTSD status were significantly correlated with recurrence (all *p* < 0.05) (Supplementary Table 8). Collinearity diagnostics were subsequently performed for variables with *p* < 0.05 in univariate analyses, and variables with acceptable multicollinearity were included in the multivariate logistic regression (Supplementary Table 9). ICU admission (OR = 1.79, 95%CI: 1.07–3.17, *p* = 0.034) and PTSD-SS score (OR = 1.02, 95%CI: 1.01–1.04, *p* < 0.001) were identified as independent influencing factors for SAP recurrence (Supplementary Table 10). After adjustment for potential confounders, ICU admission was independently associated with an increased risk of recurrence (OR = 1.84, 95% CI: 1.07–3.15, *p* = 0.027). In addition, higher PTSD-SS scores were significantly associated with an increased likelihood of recurrence (OR = 1.03, 95% CI: 1.02–1.04, *p* < 0.001) (Supplementary Table 11).

Establish mediating models to examine the mediating role of PTSD between social support and recurrence, as well as between fear of recurrence and recurrence (Supplementary Tables 12, 13). The total effect of social support on SAP recurrence was statistically significant (total effect = −0.194, *p* < 0.001). No mediating effect of PTSD was observed between social support and recurrence, nor between fear of recurrence and recurrence (all *p* > 0.05).

## 4 Discussion

This study, based on 855 patients with SAP, systematically investigated the relationships among fear of recurrence, social support, PTSD, and SAP recurrence. The findings indicated that fear of recurrence was positively associated with the occurrence of PTSD in SAP patients, whereas high levels of social support were negatively associated with PTSD and attenuated the adverse effect of fear of recurrence on PTSD. Notably, compared with PTSD status alone, PTSD severity (PTSD-SS score) independently predicted the risk of SAP recurrence. These findings suggest that psychosocial factors play an important role in the long-term prognostic management of patients with severe acute pancreatitis. Targeted psychological assessment and social support interventions for high-risk patients may contribute to improving long-term outcomes.

After experiencing a severe acute illness during the acute phase, SAP patients may continue to face uncertainty regarding disease recurrence even after completing hospitalization and surviving the life-threatening period (8). Unlike chronic diseases with relatively predictable clinical courses, SAP is characterized by an abrupt onset, rapid disease progression, and the possibility of recurrent episodes accompanied by severe complications (32). Therefore, patients may continue to worry about their future disease trajectory during the recovery phase and experience substantial disease-related psychological stress. In the present study, fear of recurrence was significantly positively correlated with PTSD-SS scores, and patients with higher levels of fear of recurrence had an increased risk of PTSD, suggesting that disease-related cognition and persistent threat perception may be important factors influencing psychological recovery in SAP patients. Persistent concerns about recurrence may lead patients to continuously monitor disease-related information, interpret normal bodily fluctuations as warning signs of recurrence, and reinforce perceptions of disease threat, thereby contributing to the persistence of post-traumatic stress symptoms (27, 33). From a Pavlovian learning perspective, repeated associations between SAP-related threatening experiences and disease-related cues may strengthen threat expectations, leading patients to interpret ambiguous bodily sensations as potential indicators of recurrence and making these fear-related beliefs more resistant to updating (34). These findings further emphasize the importance of psychological recovery after discharge among SAP patients, as focusing solely on acute-phase treatment may be insufficient to comprehensively improve long-term health outcomes.

The present study demonstrated that the severity of PTSD symptoms was independently associated with the risk of SAP recurrence, suggesting that psychological trauma responses may not only reflect previous disease experiences but may also be involved in subsequent disease recovery processes. Although this study cannot establish a causal relationship between PTSD and SAP recurrence, the two conditions may interact dynamically. On the one hand, severe disease experiences associated with SAP may trigger persistent traumatic stress responses; on the other hand, persistent PTSD symptoms may be closely associated with disease recovery processes and long-term health outcomes. Previous studies have shown that sustained psychological stress may lead to dysregulation of neuroendocrine pathways, immune dysfunction, and chronic low-grade inflammation, potentially affecting inflammatory regulation and tissue repair capacity (35, 36). In SAP patients, persistent abnormalities in inflammatory responses and immune regulation may impair pancreatic tissue recovery and overall rehabilitation processes (37), thereby potentially contributing to adverse long-term outcomes (38). In addition, PTSD-related sleep disturbances, emotional regulation difficulties, and changes in health behaviors may negatively affect treatment adherence and long-term disease management (39), potentially increasing the risk of recurrence. Therefore, PTSD may represent an important psychological factor linking traumatic disease experiences with adverse long-term health outcomes. Future studies incorporating longitudinal follow-up, inflammatory biomarkers, and behavioral assessments are needed to further clarify the specific pathways through which PTSD may influence SAP recurrence.

Pain may represent an important link between PTSD and SAP recurrence. Patients with SAP commonly experience severe abdominal pain during the acute phase, and some individuals continue to experience persistent abdominal discomfort, gastrointestinal symptoms, or intermittent pain after discharge (40) These persistent physical symptoms may continuously reinforce patients’ concerns about disease recurrence and serve as an important source of ongoing psychological stress. Previous studies have demonstrated that PTSD may amplify pain experiences by affecting central pain modulation, increasing attention toward somatic sensations, and reducing pain coping capacity (14). Pain, fear of recurrence, and PTSD may form a mutually reinforcing cycle (41): pain may increase perceptions of disease threat and promote fear of recurrence and PTSD symptoms, whereas PTSD may further enhance pain sensitivity and adversely affect recovery-related behaviors. Future studies incorporating pain assessments, inflammatory biomarkers, and neuropsychological evaluations are warranted to determine whether pain mediates the association between PTSD and SAP recurrence.

The present study also found that high levels of social support were associated with a substantially reduced risk of PTSD and moderated the association between fear of recurrence and PTSD symptoms, suggesting that social support may play an important buffering role in the psychological recovery of SAP patients. Social support theory proposes that adequate emotional support, informational support, and practical assistance can reduce individuals’ perceptions of disease-related threats, enhance coping abilities in response to stressful events, and thereby alleviate the adverse effects of persistent psychological stress on mental health (42). For patients who have experienced SAP as a severe disease event, strong social support may facilitate better understanding of the disease process, promote adaptive illness perceptions, and reduce persistent anxiety and fear caused by uncertainty regarding recurrence. Therefore, in the long-term management of SAP patients after discharge, assessment of social support levels and provision of individualized psychological support interventions for those with insufficient support may help improve psychological recovery.

This study has several limitations. First, as a single-center retrospective observational study, causal relationships cannot be established, and the single-center design may limit the generalizability of the findings. Future multicenter prospective studies are warranted to further validate our results. Second, PTSD symptoms were assessed using a self-reported screening instrument, which can evaluate symptom severity but cannot replace structured clinical interviews for diagnosis. Moreover, the single-time-point assessment may have been subject to recall bias, and previous traumatic experiences or other major stressors before SAP onset were not systematically assessed, which may have introduced residual confounding. Third, PTSD symptoms and SAP recurrence were assessed simultaneously at the 12-month follow-up, raising the possibility of reverse causality. Specifically, PTSD may increase the risk of recurrence, whereas recurrent disease experiences may also exacerbate traumatic stress responses. This temporal ambiguity limits the interpretation of the predictive role of PTSD in SAP recurrence. Furthermore, the mediation analyses were based on retrospective observational data and should therefore be interpreted as exploratory associations rather than evidence of causal mediation. Fourth, some patients were excluded due to telephone loss to follow-up or insufficient follow-up duration, and these individuals may have differed from included participants, potentially introducing selection bias. Finally, the relatively short follow-up period limited the assessment of long-term psychological adaptation and recurrence trajectories. Future studies with longer follow-up, repeated psychological assessments, and multidimensional clinical evaluations are needed to further clarify the mechanisms underlying the relationship between PTSD and SAP recurrence.

## 5 Conclusion

In conclusion, fear of recurrence significantly aggravates the severity of PTSD in SAP patients, whereas high-level social support reduces the risk of PTSD. Additionally, increased PTSD severity is independent influencing factors for SAP recurrence. In the clinical management of SAP patients, besides routine physical treatment, close attention should be paid to high-risk patients with high fear of recurrence and low social support. Targeted psychological interventions can improve patients’ traumatic stress status, reduce disease recurrence, and optimize long-term prognosis.

## Data Availability

The original contributions presented in the study are included in the article/Supplementary material, further inquiries can be directed to the corresponding author.

## References

[1] Li H Xie J Guo X Yang G Cai B Liu J . Bifidobacterium spp. and their metabolite lactate protect against acute pancreatitis via inhibition of pancreatic and systemic inflammatory responses. Gut Microbes. (2022) 14:2127456. doi: 10.1080/19490976.2022.2127456, 36195972 PMC9542615

[2] Li J Gao J Huang M Fu X Fu B. Risk factors for death in patients with severe acute pancreatitis in Guizhou Province, China. Gastroenterol Res Pract. (2024) 2024:8236616. doi: 10.1155/2024/8236616 38590392 PMC11001474

[3] Mihoc T Pirvu C Dobrescu A Brebu D Macovei AMO Pantea S . Comparative analysis of laboratory markers, severity scores, and outcomes in 179 patients with severe acute pancreatitis. Biomedicine. (2025) 13:797. doi: 10.3390/biomedicines13040797, 40299332 PMC12025027

[4] Pázmány P Kanjo A Macht-Szalai Z Gede N Farkas N Erőss B . Three-tiered critical care management of acute pancreatitis. Pancreatology. (2025) 25:39–47. doi: 10.1016/j.pan.2024.11.021, 39694759

[5] Han K Chen S Song Y Du C Gao F Liu S . Burden of pancreatitis and associated risk factors in China, 1990 to 2019: a systematic analysis for the global burden of disease study 2019. Chin Med J. (2022) 135:1340–7. doi: 10.1097/cm9.0000000000002164, 35830210 PMC9433084

[6] van Dijk SM Hallensleben NDL van Santvoort HC Fockens P van Goor H Bruno MJ . Acute pancreatitis: recent advances through randomised trials. Gut. (2017) 66:2024–32. doi: 10.1136/gutjnl-2016-313595, 28838972

[7] Møller JH Søreide K Buanes EA Kvaløy JT Strand K. Incidence, mortality, and long-term survival in patients with acute pancreatitis admitted to intensive care: a Nationwide cohort study. Acta Anaesthesiol Scand. (2026) 70:e70164. doi: 10.1111/aas.70164, 41369226

[8] Yasuda T Ueda T Takeyama Y Shinzeki M Sawa H Nakajima T . Long-term outcome of severe acute pancreatitis. J Hepato-Biliary-Pancreat Surg. (2008) 15:397–402. doi: 10.1007/s00534-007-1266-x, 18670841

[9] Bogan BD McGuire SP Maatman TK. Readmission in acute pancreatitis: etiology, risk factors, and opportunities for improvement. Surg Open Sci. (2022) 10:232–7. doi: 10.1016/j.sopen.2022.10.010, 36389270 PMC9661379

[10] Sarraf P Agrawal R Alrashdan H Agarwal M Boulay B Mutlu ER . Racial and ethnic minorities with acute pancreatitis live in neighborhoods with higher social vulnerability scores. Pancreas. (2024) 53:e317–22. doi: 10.1097/mpa.0000000000002308, 38416846 PMC10959690

[11] Righy C Rosa RG da Silva RTA Kochhann R Migliavaca CB Robinson CC . Prevalence of post-traumatic stress disorder symptoms in adult critical care survivors: a systematic review and meta-analysis. Crit Care. (2019) 23:213. doi: 10.1186/s13054-019-2489-3, 31186070 PMC6560853

[12] Jones C Bäckman C Capuzzo M Flaatten H Rylander C Griffiths RD. Precipitants of post-traumatic stress disorder following intensive care: a hypothesis generating study of diversity in care. Intensive Care Med. (2007) 33:978–85. doi: 10.1007/s00134-007-0600-8, 17384929

[13] Ma S Yang X He H Gao Y Chen Y Qin J . Psychological experience of inpatients with acute pancreatitis: a qualitative study. BMJ Open. (2022) 12:e060107. doi: 10.1136/bmjopen-2021-060107, 35768082 PMC9244672

[14] Tesarz J Baumeister D Andersen TE Vaegter HB. Pain perception and processing in individuals with posttraumatic stress disorder: a systematic review with meta-analysis. Pain Rep. (2020) 5:e849. doi: 10.1097/pr9.0000000000000849, 33490843 PMC7808684

[15] Ma S Yang X Liu Z Gao Y Zhang C Chen W . Negative emotion in patients with acute pancreatitis and its risk factors: a mixed methods systematic review. Liberation Army Nurs J. (2021) 38:6–9. doi: 10.3969/j.issn.1008-9993.2021.07.002

[16] Wang X Zhan W Huang L Guo Y Wang Y Tan H . The effect of anxiety and depression on the health-related quality of life of severe acute pancreatitis survivors: structural equation modeling approach. Front Psych. (2023) 14:1160807. doi: 10.3389/fpsyt.2023.1160807, 37200902 PMC10185751

[17] Fraile-Martinez O García-Montero C Álvarez-Mon M Casanova-Martín C Fernández-Faber D Presa M . Grasping posttraumatic stress disorder from the perspective of psychoneuroimmunoendocrinology: etiopathogenic mechanisms and relevance for integrative management. Biol Psychiatry. (2025) 98:733–45. doi: 10.1016/j.biopsych.2025.01.014, 39864788

[18] You Z Chen S Tang J. Neuroticism and posttraumatic stress disorder: a Mendelian randomization analysis. Brain Behav. (2024) 14:e70041. doi: 10.1002/brb3.70041, 39344274 PMC11440025

[19] van den Berk-Clark C Secrest S Walls J Hallberg E Lustman PJ Schneider FD . Association between posttraumatic stress disorder and lack of exercise, poor diet, obesity, and co-occuring smoking: a systematic review and meta-analysis. Health Psychol. (2018) 37:407–16. doi: 10.1037/hea0000593, 29698016 PMC5922789

[20] Menzies RE Connery T Macdonald D Riotto GD. The relationship between death anxiety and fear of recurrence and progression in chronic illness: a systematic review and meta-analysis. J Psychosom Res. (2025) 198:112373. doi: 10.1016/j.jpsychores.2025.112373, 40974620

[21] Lyhne JD Jensen LH Fink P Timm S Frostholm L Smith A. Fear of cancer recurrence in long-term colorectal cancer survivors: a nationwide cross-sectional study. J Cancer Surviv. (2025) 20:1431–42. doi: 10.1007/s11764-025-01746-z, 39871027 PMC13375672

[22] Mallahzadeh A Fereydoonnezhad T Gauthier LV Farjoud Kouhanjani M Hosseinizadeh S Khatibi A. Fear of disease progression and relapse in multiple sclerosis: a systematic scoping review. Front Psych. (2025) 16:1680781. doi: 10.3389/fpsyt.2025.1680781, 41311821 PMC12651443

[23] Paperák P Javůrková A Raudenská J. Therapeutic intervention in fear of cancer recurrence in adult oncology patients: a systematic review. J Cancer Surviv. (2023) 17:1017–35. doi: 10.1007/s11764-022-01277-x, 36307611

[24] Séguin Leclair C Lebel S Westmaas JL. The relationship between fear of cancer recurrence and health behaviors: a nationwide longitudinal study of cancer survivors. Health Psychol. (2019) 38:596–605. doi: 10.1037/hea0000754, 31120271

[25] Wenn P Meshoyrer D Barber M Ghaffar A Razka M Jose S . Perceived social support and its effects on treatment compliance and quality of life in cardiac patients. J Patient Exp. (2022) 9:23743735221074170. doi: 10.1177/23743735221074170, 35141401 PMC8819762

[26] Leifheit-Limson EC Reid KJ Kasl SV Lin H Jones PG Buchanan DM . The role of social support in health status and depressive symptoms after acute myocardial infarction: evidence for a stronger relationship among women. Circ Cardiovasc Qual Outcomes. (2010) 3:143–50. doi: 10.1161/circoutcomes.109.899815, 20160162 PMC3016989

[27] Edmondson D. An enduring somatic threat model of posttraumatic stress disorder due to acute life-threatening medical events. Soc Personal Psychol Compass. (2014) 8:118–34. doi: 10.1111/spc3.12089, 24920956 PMC4048720

[28] Banks PA Bollen TL Dervenis C Gooszen HG Johnson CD Sarr MG . Classification of acute pancreatitis--2012: revision of the Atlanta classification and definitions by international consensus. Gut. (2013) 62:102–11. doi: 10.1136/gutjnl-2012-302779, 23100216

[29] Mahendran R Liu J Kuparasundram S Griva K. Validation of the English and simplified mandarin versions of the fear of progression questionnaire—short form in Chinese cancer survivors. BMC Psychol. (2020) 8:10. doi: 10.1186/s40359-020-0374-0, 32005291 PMC6995061

[30] Qi X Wang J Liu J Amporfro DA Wang K Liu H . Factors associated with peritraumatic stress symptoms among the frontline healthcare workers during the outbreak of COVID-19 in China. BMJ Open. (2022) 12:e047753. doi: 10.1136/bmjopen-2020-047753, 35017231 PMC8753098

[31] Yang X Xue M Pauen S He H. Psychometric properties of the Chinese version of multidimensional scale of perceived social support. Psychol Res Behav Manag. (2024) 17:2233–41. doi: 10.2147/prbm.S463245, 38835653 PMC11149633

[32] Tenner S Vege SS Sheth SG Sauer B Yang A Conwell DL . American College of Gastroenterology guidelines: management of acute pancreatitis. Am J Gastroenterol. (2024) 119:419–37. doi: 10.14309/ajg.0000000000002645, 38857482 PMC13221274

[33] Lebel S Ozakinci G Humphris G Mutsaers B Thewes B Prins J . From normal response to clinical problem: definition and clinical features of fear of cancer recurrence. Support Care Cancer. (2016) 24:3265–8. doi: 10.1007/s00520-016-3272-5, 27169703

[34] Vicario CM Petrides M Tomaiuolo F Massimino S. The social life of Pavlovian learning. Cogn Affect Behav Neurosci. (2026). doi: 10.3758/s13415-026-01481-7 [Epub ahead of print]., 42521946

[35] Dell'Oste V Fantasia S Gravina D Palego L Betti L Dell'Osso L . Metabolic and inflammatory response in post-traumatic stress disorder (PTSD): a systematic review on peripheral neuroimmune biomarkers. Int J Environ Res Public Health. (2023) 20:2937. doi: 10.3390/ijerph20042937 36833633 PMC9957545

[36] Lawrence S Scofield RH. Post traumatic stress disorder associated hypothalamic-pituitary-adrenal axis dysregulation and physical illness. Brain Behav Immun Health. (2024) 41:100849. doi: 10.1016/j.bbih.2024.100849, 39280087 PMC11401111

[37] Gukovskaya AS Gukovsky I Algül H Habtezion A. Autophagy, inflammation, and immune dysfunction in the pathogenesis of pancreatitis. Gastroenterology. (2017) 153:1212–26. doi: 10.1053/j.gastro.2017.08.071, 28918190 PMC6338477

[38] Li S Gao L Gong H Cao L Zhou J Ke L . Recurrence rates and risk factors for recurrence after first episode of acute pancreatitis: a systematic review and meta-analysis. Eur J Intern Med. (2023) 116:72–81. doi: 10.1016/j.ejim.2023.06.006, 37330318

[39] Taggart Wasson L Shaffer JA Edmondson D Bring R Brondolo E Falzon L . Posttraumatic stress disorder and nonadherence to medications prescribed for chronic medical conditions: a meta-analysis. J Psychiatr Res. (2018) 102:102–9. doi: 10.1016/j.jpsychires.2018.02.013, 29631190 PMC6124486

[40] Gougol A Machicado JD Matta B Paragomi P Pothoulakis I Slivka A . Prevalence and associated factors of abdominal pain and disability at 1-year follow-up after an attack of acute pancreatitis. Pancreas. (2019) 48:1348–53. doi: 10.1097/mpa.0000000000001434, 31688600 PMC6839779

[41] Ravyts SG Hall R Vandine D Woods O Harris R Dudeney J . PTSD and chronic pain: a systematic review and meta-analysis of mediation studies. J Pain. (2026) 38:105589. doi: 10.1016/j.jpain.2025.105589, 41207404

[42] Cohen S Wills TA. Stress, social support, and the buffering hypothesis. Psychol Bull. (1985) 98:310–57. doi: 10.1037/0033-2909.98.2.310, 3901065

