# Supplementary material for: Associations of fear of recurrence and social support with post-traumatic stress disorder and disease recurrence in patients with severe acute pancreatitis: a retrospective study
Source: Front Med (Lausanne). 2026 Aug 19;13:1893298. doi: 10.3389/fmed.2026.1893298 (PMC13533815; doi:10.3389/fmed.2026.1893298)
Supplement: Supplementary file 1 [file Supplementary_file_1.docx]

Supplementary Table 1 Univariate Binary Logistic Regression Analysis of Influencing Factors for PTSD Development in Patients with Severe Acute Pancreatitis

| **Characteristic** | **OR** | **95% CI** | ***P*-value** |
| --- | --- | --- | --- |
| **Education** |  |  |  |
| Primary school or below | Reference | |  |
| Middle or high school | 1.31 | 0.91, 1.90 | 0.147 |
| College or undergraduate | 1.16 | 0.78, 1.74 | 0.457 |
| Master's degree or above | 1.81 | 0.45, 6.57 | 0.369 |
| Missing | 0.68 | 0.03, 4.71 | 0.733 |
| **Use of psychotropic drugs** |  |  |  |
| No | Reference | |  |
| Yes | 0.00 |  | 0.970 |
| **Alcohol cessation status** |  |  |  |
| Never drinker | Reference | |  |
| Former drinker | 1.14 | 0.68, 1.87 | 0.604 |
| Current drinker | 0.96 | 0.70, 1.32 | 0.814 |
| Heavy drinker | 0.33 | 0.02, 1.86 | 0.297 |
| **Smoking cessation status** |  |  |  |
| Never smoker | Reference | |  |
| Former smoker | 0.75 | 0.42, 1.28 | 0.312 |
| Current smoker | 1.07 | 0.78, 1.47 | 0.679 |
| **Sex** |  |  |  |
| Female | Reference | |  |
| Male | 1.04 | 0.76, 1.43 | 0.798 |
| **Marital Status** |  |  |  |
| Divorced | Reference | |  |
| Married | 1.40 | 0.65, 3.37 | 0.411 |
| Missing | 1.95 | 0.34, 9.91 | 0.424 |
| Unmarried | 1.67 | 0.71, 4.27 | 0.257 |
| Widowed | 0.93 | 0.22, 3.52 | 0.915 |
| **Age** | 1.00 | 0.99, 1.01 | 0.384 |
| **Career** |  |  |  |
| Employed | Reference | |  |
| Unemployed | 0.96 | 0.68, 1.35 | 0.805 |
| Retired | 0.98 | 0.58, 1.65 | 0.946 |
| **Place of Residence** |  |  |  |
| Urban | Reference | |  |
| Rural | 0.89 | 0.66, 1.19 | 0.430 |
| **Hypertension** |  |  |  |
| No | Reference | |  |
| Yes | 0.94 | 0.67, 1.31 | 0.727 |
| **Diabetes Mellitus** |  |  |  |
| No | Reference | |  |
| Yes | 1.22 | 0.89, 1.66 | 0.216 |
| **Hyperlipidemia** |  |  |  |
| No | Reference | |  |
| Yes | 0.95 | 0.67, 1.32 | 0.740 |
| **Coronary Heart Disease** |  |  |  |
| No | Reference | |  |
| Yes | 0.82 | 0.26, 2.16 | 0.699 |
| **Cancer** |  |  |  |
| No | Reference | |  |
| Yes | 1.48 | 0.54, 3.79 | 0.433 |
| **History of Surgery** |  |  |  |
| No | Reference | |  |
| Yes | 0.85 | 0.59, 1.19 | 0.344 |
| **History of Major Diseases** |  |  |  |
| No | Reference | |  |
| Yes | 0.59 | 0.23, 1.29 | 0.193 |
| **Etiology** |  |  |  |
| Alcoholic | Reference | |  |
| Biliary | 0.80 | 0.30, 2.41 | 0.677 |
| Hypertriglyceridemic | 0.80 | 0.30, 2.36 | 0.667 |
| missing | 0.67 | 0.03, 6.60 | 0.747 |
| Other | 0.99 | 0.37, 2.91 | 0.985 |
| **Organ Failure** |  |  |  |
| No | Reference | |  |
| Yes | 1.04 | 0.77, 1.40 | 0.812 |
| **ICU Admission** |  |  |  |
| No | Reference | |  |
| Yes | 1.14 | 0.79, 1.68 | 0.484 |
| **Invasive Mechanical Ventilation** |  |  |  |
| No | Reference | |  |
| Yes | 0.80 | 0.57, 1.11 | 0.187 |
| **Surgical Treatment** |  |  |  |
| No | Reference | |  |
| Yes | 0.98 | 0.70, 1.39 | 0.912 |
| **FoP-Q-SF score** | 1.56 | 1.46, 1.69 | <0.001 |
| **Fear of Recurrence** | 39.5 | 21.8, 90.3 | <0.001 |
| **PSSS score** | 0.71 | 0.66, 0.75 | <0.001 |
| **Perceived social support** |  |  |  |
| Low mid support | Reference | |  |
| High support | 0.00 | 0.00, 0.00 | <0.001 |

History of serious diseases including Acute myocardial infarction, cardiogenic shock, stroke, major trauma; PTSD, Post-traumatic Stress Disorder; OR, Odds ratio; CI, Confidence interval; ICU, Intensive Care Unit; FoP-Q-SF, Fear of Progression Questionnaire-Short Form; PSSS, Perceived Social Support Scale.

Supplementary Table 2 Univariate Linear Regression Analysis of Influencing Factors for PTSD-SS Score in Patients with Severe Acute Pancreatitis.

| **Characteristic** | **β** | **95% CI** | ***P*-value** |
| --- | --- | --- | --- |
| **Education** |  |  |  |
| Primary school or below | Reference | |  |
| Middle or high school | 4.123 | -0.074, 8.321 | 0.054 |
| College or undergraduate | 1.480 | -3.089, 6.048 | 0.525 |
| Master's degree or above | 5.474 | -10.579, 21.528 | 0.503 |
| Missing | 2.374 | -20.088, 24.837 | 0.836 |
| **Use of psychotropic drugs** |  |  |  |
| No | Reference | |  |
| Yes | -18.190 | -38.523, 2.142 | 0.079 |
| **Alcohol cessation status** |  |  |  |
| Never drinker | Reference | |  |
| Former drinker | -0.932 | -6.904, 5.040 | 0.759 |
| Current drinker | 0.464 | -3.270, 4.197 | 0.807 |
| Heavy drinker | -5.383 | -23.120, 12.355 | 0.552 |
| **Smoking cessation status** |  |  |  |
| Never smoker | Reference | |  |
| Former smoker | -2.640 | -8.725, 3.446 | 0.395 |
| Current smoker | 0.844 | -2.879, 4.566 | 0.657 |
| **Sex** |  |  |  |
| Female | Reference | |  |
| Male | 0.494 | -3.143, 4.130 | 0.790 |
| **Marital Status** |  |  |  |
| Divorced | Reference | |  |
| Married | 3.292 | -5.435, 12.019 | 0.459 |
| Missing | 17.485 | -2.032, 37.003 | 0.079 |
| Unmarried | 6.350 | -3.407, 16.107 | 0.202 |
| Widowed | 0.791 | -13.687, 15.269 | 0.915 |
| **Age** | -0.052 | -0.157, 0.052 | 0.326 |
| **Career** |  |  |  |
| Employed | Reference | |  |
| Unemployed | -0.323 | -4.302, 3.656 | 0.873 |
| Retired | -0.859 | -6.966, 5.249 | 0.783 |
| **Place of Residence** |  |  |  |
| Urban | Reference | |  |
| Rural | -1.325 | -4.729, 2.078 | 0.445 |
| **Hypertension** |  |  |  |
| No | Reference | |  |
| Yes | -0.796 | -4.697, 3.105 | 0.689 |
| **Diabetes Mellitus** |  |  |  |
| No | Reference | |  |
| Yes | 1.890 | -1.785, 5.564 | 0.313 |
| **Hyperlipidemia** |  |  |  |
| No | Reference | |  |
| Yes | -2.636 | -6.516, 1.244 | 0.183 |
| **Coronary Heart Disease** |  |  |  |
| No | Reference | |  |
| Yes | -3.877 | -15.409, 7.655 | 0.510 |
| **Cancer** |  |  |  |
| No | Reference | |  |
| Yes | 4.079 | -7.762, 15.920 | 0.499 |
| **History of Surgery** |  |  |  |
| No | Reference | |  |
| Yes | -2.939 | -6.910, 1.032 | 0.147 |
| **History of Major Diseases** |  |  |  |
| No | Reference | |  |
| Yes | -1.454 | -10.155, 7.247 | 0.743 |
| **Etiology** |  |  |  |
| Alcoholic | Reference | |  |
| Biliary | -11.268 | -23.534, 0.998 | 0.072 |
| Hypertriglyceridemic | -11.091 | -23.101, 0.919 | 0.070 |
| missing | -14.861 | -42.322, 12.600 | 0.288 |
| Other | -9.114 | -21.168, 2.939 | 0.138 |
| **Organ Failure** |  |  |  |
| No | Reference | |  |
| Yes | -0.189 | -3.692, 3.313 | 0.915 |
| **ICU Admission** |  |  |  |
| No | Reference | |  |
| Yes | 0.558 | -3.761, 4.876 | 0.800 |
| **Invasive Mechanical Ventilation** |  |  |  |
| No | Reference | |  |
| Yes | -2.095 | -5.943, 1.752 | 0.285 |
| **Surgical Treatment** |  |  |  |
| No | Reference | |  |
| Yes | -0.221 | -4.266, 3.824 | 0.915 |
| **FoP-Q-SF score** | 1.605 | 1.507, 1.704 | <0.001 |
| **Fear of Recurrence** | 13.507 | 12.773, 14.241 | <0.001 |
| **PSSS score** | -1.072 | -1.144, -0.999 | <0.001 |
| **Perceived social support** |  |  |  |
| Low mid support | Reference | |  |
| High support | -45.223 | -47.355, -43.090 | <0.001 |

History of serious diseases including Acute myocardial infarction, cardiogenic shock, stroke, major trauma; PTSD-SS, Post-traumatic Stress Disorder self-rating scale; CI, Confidence interval; ICU, Intensive Care Unit; FoP-Q-SF, Fear of Progression Questionnaire-Short Form; PSSS, Perceived Social Support Scale.

Supplementary Table 3 Multicollinearity Diagnostics for multivariate logistic regression of PTSD Development.

| **Variable** | **VIF** | **Tolerance** |
| --- | --- | --- |
| **FoP-Q-SF score** | 1.828 | 0.547 |
| **Fear of Recurrence** | 2.874 | 0.348 |
| **PSSS score** | 1.649 | 0.607 |
| **Perceived social support** | 1.859 | 0.538 |

PTSD, Post-traumatic Stress Disorder; VIF, variance inflation factor; FoP-Q-SF, Fear of Progression Questionnaire-Short Form; PSSS, Perceived Social Support Scale.

Supplementary Table 4 Multicollinearity Diagnostics for multivariate linear regression of PTSD-SS Score.

| **Variable** | **VIF** | **Tolerance** |
| --- | --- | --- |
| **FoP-Q-SF score** | 4.820 | 0.207 |
| **Fear of Recurrence** | 7.430 | 0.135 |
| **PSSS score** | 3.517 | 0.284 |
| **Perceived social support** | 11.461 | 0.087 |

PTSD-SS, Post-traumatic Stress Disorder self-rating scale; VIF, variance inflation factor; FoP-Q-SF, Fear of Progression Questionnaire-Short Form; PSSS, Perceived Social Support Scale.

Supplementary Table 5 Multivariate Binary Logistic Regression Analysis of Independent Influencing Factors for PTSD Development in Patients with Severe Acute Pancreatitis

| **Characteristic** | **OR** | **95% CI** | ***P*-value** |
| --- | --- | --- | --- |
| **Fear of recurrence** | 3.67 | 1.35, 12.4 | 0.019 |
| **Perceived social support** |  |  |  |
| low mid support | Reference | |  |
| high support | 0.05 | 0.01, 0.45 | 0.002 |
| **PSSS score** | 0.96 | 0.90, 1.02 | 0.163 |
| **FoP-Q-SF score** | 1.09 | 0.99, 1.20 | 0.070 |

PTSD, Post-traumatic Stress Disorder; OR, Odds ratio; CI, Confidence interval; PSSS, Perceived Social Support Scale; FoP-Q-SF, Fear of Progression Questionnaire-Short Form.

Supplementary Table 6 Analysis of the interaction effect between fear of recurrence and perceived social support on PTSD in patients with severe acute pancreatitis.

| **Characteristic** | **OR** | **95% CI** | ***P*-value** |
| --- | --- | --- | --- |
| **Fear of recurrence** | 1,415.142 | 22.766, 578,587.813 | 0.003 |
| **PSSS score** | 1.055 | 0.968, 1.165 | 0.246 |
| **Perceived social support** |  |  |  |
| low mid support | Reference | |  |
| High support | 0.155 | 0.012, 23.728 | 0.265 |
| **FoP-Q-SF score** | 1.024 | 0.908, 1.139 | 0.676 |
| **Fear of recurrence * PSSS score** | 0.889 | 0.805, 0.957 | 0.005 |

PTSD, Post-traumatic Stress Disorder; OR, Odds ratio; CI, Confidence interval; PSSS, Perceived Social Support Scale; FoP-Q-SF, Fear of Progression Questionnaire-Short Form.

Supplementary Table 7 Multivariate Linear Regression and Interaction Effect Analysis of Influencing Factors for PTSD-SS Score in Patients with Severe Acute Pancreatitis

| **Characteristic** | **β** | **95% CI** | ***P*-value** |
| --- | --- | --- | --- |
| PSSS score | -0.500 | -0.604, -0.397 | <0.001 |
| FoP-Q-SF score | 1.052 | 0.904, 1.200 | <0.001 |

PTSD-SS, Post-traumatic Stress Disorder self-rating scale; CI, Confidence interval; PSSS, Perceived Social Support Scale; FoP-Q-SF, Fear of Progression Questionnaire-Short Form.

Supplementary Table 8 Univariate Logistic Regression Analysis of Influencing Factors for Recurrence in Patients with Severe Acute Pancreatitis

| **Characteristic** | **OR** | **95% CI** | ***P*-value** |
| --- | --- | --- | --- |
| **Education** |  |  |  |
| Primary school or below | Reference | |  |
| Middle or high school | 0.93 | 0.60, 1.44 | 0.726 |
| College or undergraduate | 0.85 | 0.52, 1.38 | 0.510 |
| Master's degree or above | 1.13 | 0.17, 4.73 | 0.876 |
| Missing | 0.00 |  | 0.983 |
| **Use of psychotropic drugs** |  |  |  |
| No | Reference | |  |
| Yes | 0.00 |  | 0.981 |
| **Alcohol cessation status** |  |  |  |
| Never drinker | Reference | |  |
| Former drinker | 0.64 | 0.30, 1.23 | 0.207 |
| Current drinker | 0.87 | 0.58, 1.28 | 0.474 |
| Heavy drinker | 0.65 | 0.03, 3.70 | 0.685 |
| **Smoking cessation status** |  |  |  |
| Never smoker | Reference | |  |
| Former smoker | 0.44 | 0.18, 0.92 | 0.046 |
| Current smoker | 0.83 | 0.56, 1.22 | 0.348 |
| **Sex** |  |  |  |
| Female | Reference | |  |
| Male | 1.02 | 0.70, 1.51 | 0.925 |
| **Marital Status** |  |  |  |
| Divorced | Reference | |  |
| Married | 1.16 | 0.48, 3.45 | 0.770 |
| Missing | 3.48 | 0.57, 19.7 | 0.155 |
| Unmarried | 1.30 | 0.48, 4.19 | 0.626 |
| Widowed | 0.73 | 0.10, 3.80 | 0.719 |
| **Age** | 1.00 | 0.99, 1.01 | 0.756 |
| **Career** |  |  |  |
| Employed | Reference | |  |
| Unemployed | 0.96 | 0.64, 1.48 | 0.860 |
| Retired | 1.24 | 0.66, 2.26 | 0.498 |
| **Place of Residence** |  |  |  |
| Urban | Reference | |  |
| Rural | 0.86 | 0.60, 1.23 | 0.405 |
| **Hypertension** |  |  |  |
| No | Reference | |  |
| Yes | 0.97 | 0.63, 1.45 | 0.880 |
| **Diabetes Mellitus** |  |  |  |
| No | Reference | |  |
| Yes | 0.94 | 0.63, 1.38 | 0.746 |
| **Hyperlipidemia** |  |  |  |
| No | Reference | |  |
| Yes | 0.75 | 0.48, 1.14 | 0.187 |
| **Coronary Heart Disease** |  |  |  |
| No | Reference | |  |
| Yes | 0.57 | 0.09, 2.04 | 0.431 |
| **Cancer** |  |  |  |
| No | Reference | |  |
| Yes | 0.00 |  | 0.010 |
| **History of Surgery** |  |  |  |
| No | Reference | |  |
| Yes | 0.84 | 0.54, 1.28 | 0.425 |
| **History of Major Diseases** |  |  |  |
| No | Reference | |  |
| Yes | 0.47 | 0.11, 1.33 | 0.169 |
| **Etiology** |  |  |  |
| Alcoholic | Reference | |  |
| Biliary | 1.93 | 0.52, 12.6 | 0.394 |
| Hypertriglyceridemic | 1.61 | 0.44, 10.4 | 0.532 |
| missing | 0.00 | 0.00, 0.00 | 0.977 |
| Other | 1.51 | 0.41, 9.76 | 0.590 |
| **Organ Failure** |  |  |  |
| No | Reference | |  |
| Yes | 1.05 | 0.72, 1.51 | 0.812 |
| **ICU Admission** |  |  |  |
| No | Reference | |  |
| Yes | 1.81 | 1.09, 3.15 | 0.020 |
| **Invasive Mechanical Ventilation** |  |  |  |
| No | Reference | |  |
| Yes | 1.08 | 0.72, 1.60 | 0.715 |
| **Surgical Treatment** |  |  |  |
| No | Reference | |  |
| Yes | 1.28 | 0.83, 2.04 | 0.269 |
| **FoP-Q-SF score** | 1.05 | 1.03, 1.07 | <0.001 |
| **Fear of Recurrence** | 1.50 | 1.34, 1.69 | <0.001 |
| **PSSS score** | 0.97 | 0.96, 0.98 | <0.001 |
| **Perceived social support** |  |  |  |
| Low mid support | Reference | |  |
| High support | 0.28 | 0.19, 0.41 | <0.001 |
| **PTSD** |  |  |  |
| No PTSD | Reference | |  |
| PTSD | 3.59 | 2.48, 5.20 | <0.001 |
| **PTSD severity** |  |  |  |
| Mild PTSD | Reference | |  |
| Moderate to severe PTSD | 3.16 | 0.55, 59.50 | 0.286 |
| No PTSD | 0.86 | 0.15, 16.10 | 0.886 |

History of serious diseases including Acute myocardial infarction, cardiogenic shock, stroke, major trauma; OR, Odds ratio; CI, Confidence interval; ICU, Intensive Care Unit; PTSD, Post-traumatic Stress Disorder; PSSS, Perceived Social Support Scale; FoP-Q-SF, Fear of Progression Questionnaire-Short Form; PTSD-SS, Post-traumatic Stress Disorder self-rating scale.

Supplementary Table 9 Multicollinearity Diagnostics for multivariate logistic regression of Post-discharge Recurrence.

| **Variable** | **GVIF** | **Degrees of freedom** | **Adjusted GVIF** |
| --- | --- | --- | --- |
| **Smoking cessation status** | 1.017 | 2 | 1.004 |
| **ICU admission** | 1.005 | 1 | 1.003 |
| **FoP-Q-SF score** | 7.112 | 1 | 2.667 |
| **Fear of Recurrence** | 9.463 | 1 | 3.076 |
| **PSSS score** | 4.729 | 1 | 2.175 |
| **Perceived social support** | 36.815 | 1 | 6.068 |
| **PTSD-SS** | 3.662 | 1 | 1.914 |
| **PTSD** | 22.992 | 1 | 4.795 |

GVIF, Generalized Variance Inflation Factor; ICU, Intensive Care Unit; FoP-Q-SF, Fear of Progression Questionnaire-Short Form; PSSS, Perceived Social Support Scale; PTSD-SS, Post-traumatic Stress Disorder self-rating scale; PTSD, Post-traumatic Stress Disorder.

Supplementary Table 10 Multivariate Logistic Regression and Interaction of Influencing Factors for Post-discharge Recurrence in Patients with Severe Acute Pancreatitis

| **Characteristic** | **OR** | **95% CI** | ***P-*value** |
| --- | --- | --- | --- |
| **Smoking cessation status** |  |  |  |
| Never smoker | Reference | |  |
| Former smoker | 0.48 | 0.19, 1.03 | 0.082 |
| Current smoker | 0.81 | 0.54, 1.21 | 0.314 |
| **ICU admission** |  |  |  |
| No | Reference | |  |
| Yes | 1.79 | 1.07, 3.17 | 0.034 |
| **PSSS score** | 0.99 | 0.98, 1.01 | 0.375 |
| **PTSD-SS score** | 1.02 | 1.01, 1.04 | <0.001 |

OR, Odds ratio; CI, Confidence interval; ICU, Intensive Care Unit; PTSD, PSSS, Perceived Social Support Scale; PTSD-SS, Post-traumatic Stress Disorder self-rating scale.

Supplementary Table 11 Multivariate Logistic Regression Analysis of the Effects of Fear of Recurrence, Perceived Social Support, and Their Interaction on Recurrence in Patients with Severe Acute Pancreatitis

| **Analysis** | **Characteristic** | **OR** | **95% CI** | ***P*-value** |
| --- | --- | --- | --- | --- |
| ^1^Main model | ICU admission, Yes vs No | 1.84 | 1.07, 3.15 | 0.027 |
|  | PTSD-SS score | 1.03 | 1.02, 1.04 | <0.001 |
| Interaction analysis | ^2^PTSD-SS score × Fear of recurrence | 1.00 | 0.99, 1.01 | 0.719 |
|  | ^3^PTSD-SS score × PSSS score | 1.00 | 1.00, 1.00 | 0.222 |

^1^In main model, ICU admission and PTSD-SS score were adjusted; ^2^in interaction analysis, ICU admission and interaction term of PTSD-SS score and fear of recurrence were adjusted in PTSD-SS score × Fear of recurrence; ^3^ICU admission and interaction term of PTSD-SS score and PSSS score were adjusted in PTSD-SS score × PSSS score. OR, Odds ratio; CI, Confidence interval; ICU, Intensive Care Unit; PTSD-SS, Post-traumatic Stress Disorder self-rating scale; PSSS, Perceived Social Support Scale.

Supplementary Table 12 Mediating Effect of PTSD Between Social Support and Recurrence

| **Metric** | **Estimate** | **95% CI** | ***P*-value** |
| --- | --- | --- | --- |
| ACME (average) | -0.128 | (-0.306, 0.043) | 0.150 |
| ADE (average) | -0.067 | (-0.253, 0.117) | 0.372 |
| Total Effect | -0.194 | (-0.255, -0.132) | <0.001 |
| Proportion mediated (average) | 0.657 | (-0.240, 1.727) | 0.150 |

PTSD, Post-traumatic Stress Disorder; CI, Confidence interval; ACME, Average Causal Mediating Effect; ADE, Average Direct Effect.

Supplementary Table 13 Mediating Effect of PTSD Between Fear of Recurrence and Recurrence

| **Metric** | **Estimate** | **95% CI** | ***P*-value** |
| --- | --- | --- | --- |
| ACME (average) | 0.003 | (-0.001, 0.009) | 0.180 |
| ADE (average) | 0.020 | (-0.008, 0.049) | 0.124 |
| Total Effect | 0.024 | (-0.002, 0.049) | 0.062 |
| Proportion mediated (average) | 0.144 | (-0.590, 1.349) | 0.242 |

PTSD, Post-traumatic Stress Disorder; CI, Confidence interval; ACME, Average Causal Mediating Effect; ADE, Average Direct Effect
